# The feasibility of laparoscopic TSME preserving the left colic artery and superior rectal artery for upper rectal cancer

**DOI:** 10.1186/s12957-020-01986-6

**Published:** 2020-08-18

**Authors:** Chi Zhang, Hao-tang Wei, Wenqing Hu, Yueming Sun, Qinyuan Zhang, Masanobu Abe, Zhuoran Du, Yingying Xu, Liang Zong, Xiang Hu

**Affiliations:** 1 Department of Gastrointestinal Surgery, The First Affiliated Hospital of Dalian Medical University, Dalian, Liaoning Province China; 2 Department of Gastrointestinal Surgery, The Third Affiliated Hospital of Guangxi Medical University, Nanning, Guangxi Province China; 3 Department of Gastrointestinal Surgery, Changzhi People’s Hospital, The Affiliated Hospital of Changzhi Medical College, Changzhi, Shanxi Province China; 4 Department of Colorectal Surgery, The First Affiliated Hospital of Nanjing Medical University, Nanjing, China; 5 Department of Gastrointestinal Surgery, The First People’s Hospital of Dali City, Dali, Yunnan Province China; 6 Department of Gastrointestinal Surgery, Graduate School of Medicine, University of Tokyo, Tokyo, Japan; 7 Department of General Surgery, Yizhen People’s Hospital, Clinical Medical College, Yangzhou University, Yangzhou, Jiangsu Province China

**Keywords:** Laparoscopic surgery, Rectal cancer, Tumor-specific mesorectal excision, Superior rectal artery, Left colonic artery, TME

## Abstract

**Background:**

Laparoscopic tumor-specific mesorectal excision (TSME) preserving the left colic artery and superior rectal artery is still a technically challenging procedure. We conducted this study to demonstrate the feasibility of this procedure for upper rectal cancer.

**Methods:**

A total of 184 patients with upper rectal cancer were retrospectively analyzed in our cancer center between April 2010 and April 2017. These patients were treated with either laparoscopic TSME (*n* = 46) or laparoscopic total mesorectal excision (TME) (*n* = 138). In the TSME group, the left colonic artery and superior rectal artery were preserved while they were not in the TME group.

**Results:**

The operation time in the TSME group was longer than that in the TME group (218.56 ± 35.85 min vs. 201.13 ± 42.65 min, *P* = 0.004). Furthermore, the number of resected lymph nodes in the TSME group was greater than that in the TME group (19.43 ± 9.46 vs. 18.03 ± 7.43, *P* = 0.024). The blood loss between the TSME and TME groups was not significant. No mortality occurred in either the TSME or TME groups. One patient in the TME group underwent conversion to laparotomy. The total postoperative complication rates in the TSME and TME groups were 8.7% and 17.4%, respectively. There was no difference in severe complications between the two groups (anastomotic leakage and stenosis).

**Conclusions:**

Laparoscopic TSME preserving the left colic artery and superior rectal artery can be safely conducted for upper rectal cancer.

## Introduction

Total mesorectal excision (TME) is an important surgical technique to prevent the local recurrence of rectal cancer [1]. On the other hand, TME may not be suitable for every case of rectal cancer, such as rectosigmoid junction and upper rectal cancers. The resection range of TME reaches 5 cm below the inferior border of the tumor and has acquired an adequate cure rate reported in previous studies for patients with rectosigmoid junction and upper rectal cancers [2]. This tumor-specific resection according to the tumor site or T staging is called tumor-specific mesorectal excision (TSME) [3].

Sudeck’s critical point at the rectosigmoid junction is described as the point of origin of the last sigmoid arterial branch, originating from the inferior mesenteric artery (IMA) [4]. The anastomosis between the last sigmoidal artery and superior rectal artery (SRA) is absent in some people. To avoid the risk of postoperative ischemic necrosis, anastomotic leakage, colitis, and delayed stricture, it is desirable to ligate proximal to Sudeck’s point, for cases where anastomosis may be absent or insufficiently present [5]. In addition, the rate of absence of the left colic artery (LCA) is 1.2%, which may be associated with a risk of anastomotic leakage due to insufficient vascularization of the proximal colonic conduit [6].

This study introduces the procedure and technical points of laparoscopic TSME with preservation of the LCA and SRA. The operation is still a technically challenging procedure. We conducted this study to demonstrate the feasibility of this procedure for upper rectal cancer and short-term prognosis.

## Methods

### Patients

Laparoscopic TSME preserving the LCA and SRA was performed on 46 patients with upper rectal cancer from April 2010 to April 2017. In the same period, 138 patients with upper rectal cancer underwent standard TME surgery. This study was conducted in accordance with approved guidelines. This study was approved by the Institutional Review Board of the First Affiliated Hospital of Dalian Medical University. Written informed consent was obtained from all patients.

### Equipment

Angled (30°) 10-mm diameter 3D laparoscope, insufflation equipment, and bipolar electrosurgical device (Aesculap German); harmonic vascular closure system (Johnson USA); 10-mm and 5-mm port trocars (Teleflex Medical, USA); laparoscopic linear staplers (60 mm in length, COVIDIEN USA); hem-o-lock polymer locking surgical clips (Teleflex Medical, USA); and a circular stapler (ETHICON Endosurgery, USA) were used in this study.

### Preoperative preparation

**Fig. 1:**
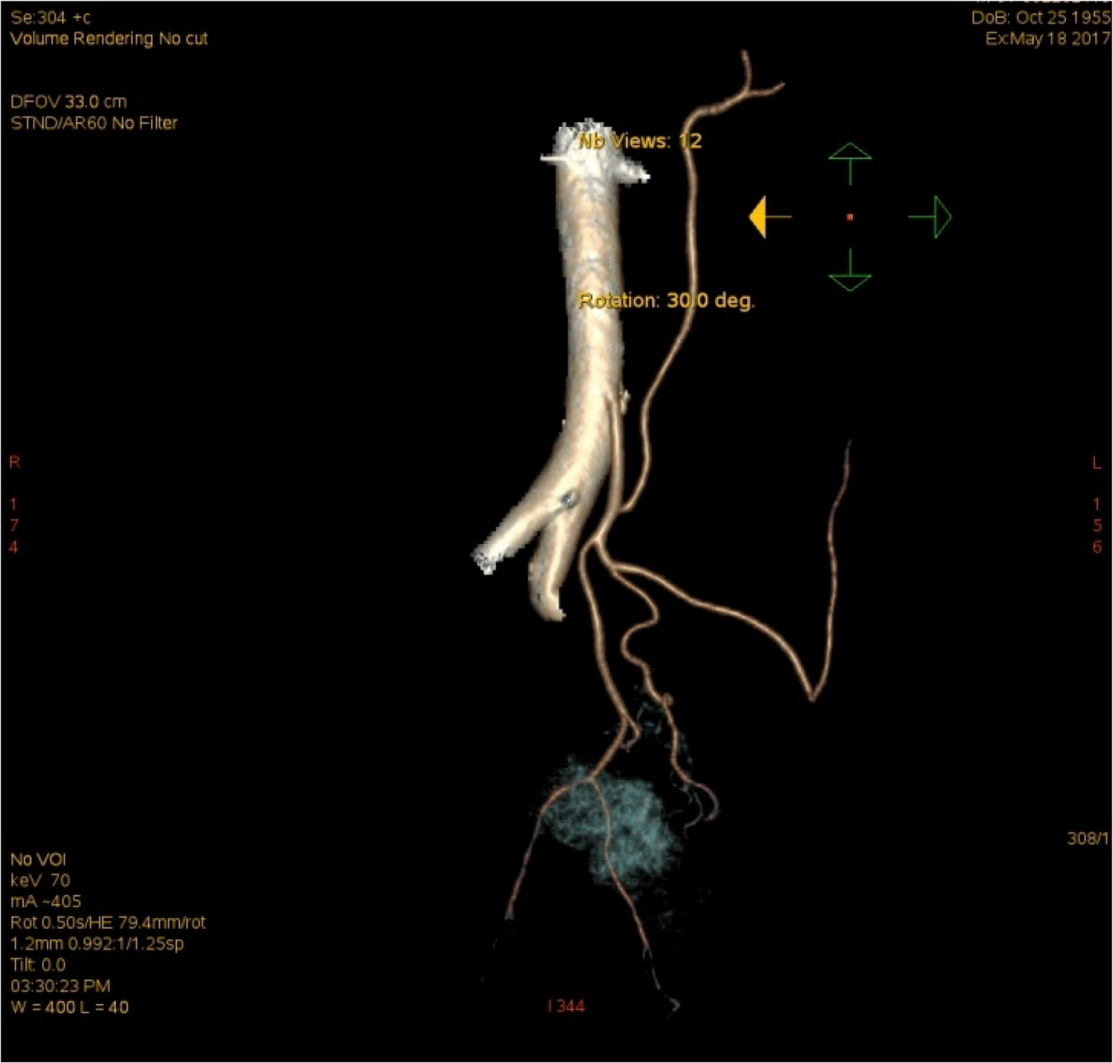
IMA 3D CT-A

Inferior mesenteric artery (IMA) 3D CT-A examination should be performed before the operation to assess the mesenteric vascular vessel types (Fig. 1). Intestinal preparation was performed 2 days before the operation, and prophylactic intravenous antibiotics were used before the operation for 30 min. Central venous catheterization was performed after general anesthesia. The surgical posture was the starboard lithotomy position with the head lower and feet higher.

**Fig. 2:**
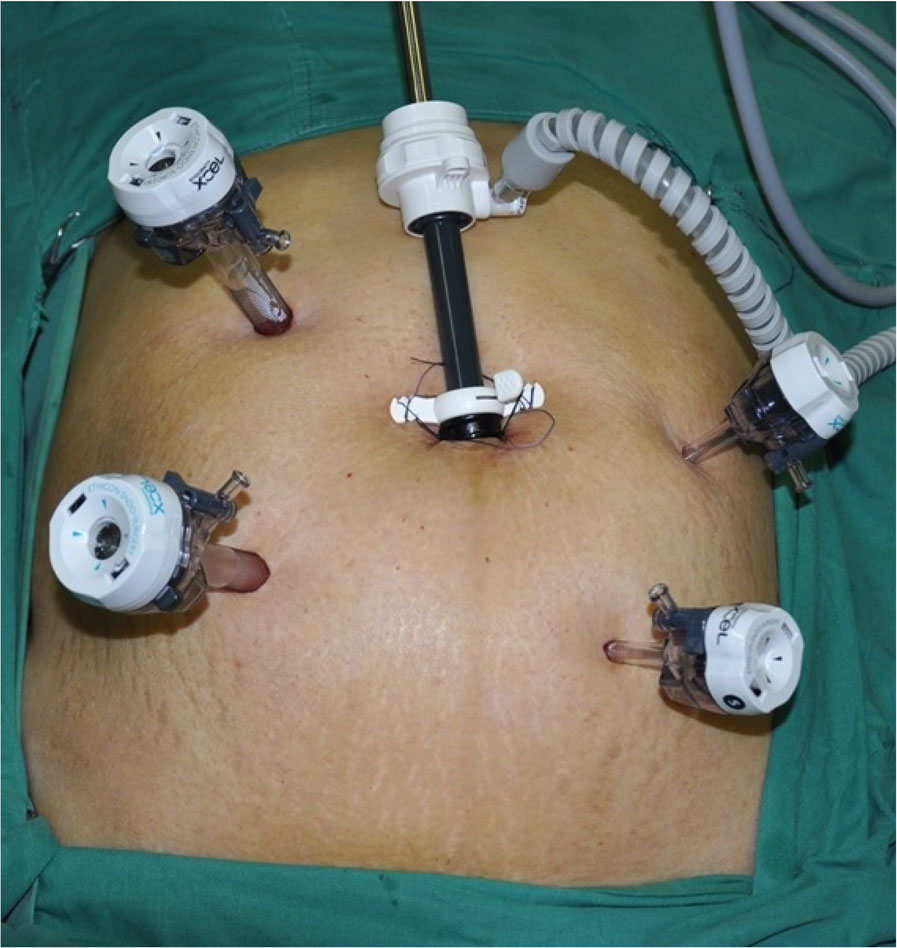
Position of the trocar

The operating surgeon and camera assistant stood on the patient’s right side, and the first assistant stood at the patient’s left foot side. The laparoscopic monitor was placed on the patient’s right foot side. The trocar for the laparoscope was inserted from the right paraumbilical side, and four ports were used as working ports (Fig. 2).

### Surgical techniques

This surgical technique was characterized by thorough lymph node dissection based on neurovascular preservation and dissection of the left colon and sigmoid and upper rectal vessels along the inferior mesenteric vessels. The region of operation was the superficial layer of the nerve sheath on the vascular surface. The left colonic and superior rectal vessels needed to be preserved, and the vascular branch from the sigmoid vessels and the blood vessels from the superior rectal vessels to the intestinal wall were selected and severed according to the tumor position.

First, we adopted a lateral approach by opening the monks’ white line along the descending and sigmoid colon reaching the splenic flexure as the cephalad dissection point. The correct plane of dissection was achieved by Toldt’s fascia. We usually used bipolar electrosurgical devices and bipolar scissors to separate this correct plane with gentle blunt and sharp dissection. The ureter and other retroperitoneal structures were safely protected by staying in this plane. We continued to dissect along the plane to the root of the IMA. The hypogastric nerves were visible. The nerves were carefully protected.

**Fig. 3:**
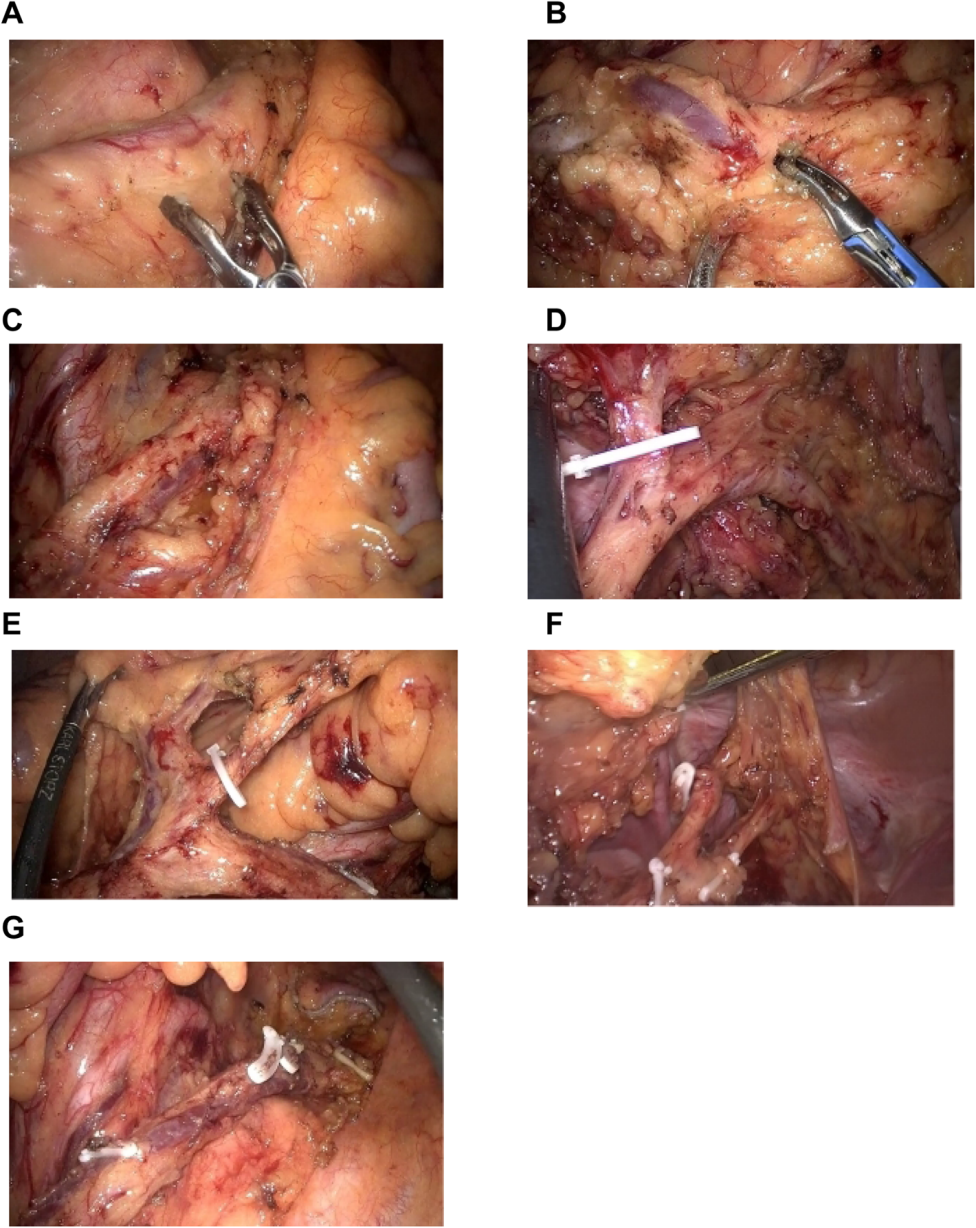
**a** Dissection the presacral space below the superior rectal artery (SRA) approached from the left side across the midline to the right side attentively protected hypogastric nerves while using a bipolar electrosurgical device. **b** Identification of the relationship between left colic artery/IMV to the IMA and SRA and the branch of the arteriae sigmoideae. **c** Tracing this vascular bundle from the origin of the IMA to the rectum segment approximately 4–5 cm below the inferior border of the tumor. **d** Ligation of arteriae sigmoideae and vascular branch from SRA. **e** Ligation of arteriae sigmoideae and preserving left colonic vasculature. **f** Excision of the mesorectum just underneath the rectal wall about 3–5 cm and avoiding injury to the rectal wall and SRA. **g** TSME preserving left colic artery and superior rectal artery

Then, the dissection began at the position of the sacral promontory, the junction of the sigmoid mesentery and retroperitoneum from the previous dissection plane in the first step. Ideally, we dissected the presacral space below the SRA from the left side across the midline to the right side, attentively protecting the hypogastric nerves while using a bipolar electrosurgical device (Fig. 3a). The distal dissection endpoint was approximately 4–5 cm below the tumor. We needed to open the peritoneal reflection and dissect the lateral ligament of the rectum by protecting the neurovascular bundle (NVB) using a harmonic vascular closure system in some patients. We placed the dissected colon and mesocolon to the right celiac side and thoroughly revealed the left side of the mesocolon. We carefully employed dissection in the correct plane on the vessels to avoid tissue damage for the realization of en bloc resection. The technique in this step is to identify the relationship between the left colic artery inferior mesenteric vein (IMV) to the IMA and SRA and the branch of the arteriae sigmoideae (Fig. 3b). This vascular bundle can be traced from the origin of the IMA to the rectal segment approximately 4–5 cm below the inferior border of the tumor (Fig. 3c).

The second step was performed using a medial approach. This step involved thorough lymph node dissection based on neurovascular preservation. The left colonic and superior rectal vessels need to be preserved, and the sigmoid vessels and vessel branch from the superior rectal vessels to the intestinal wall were selected and severed according to the tumor position.

Dissection at the correct presacral space and cephalad dissection to the IMA could be employed. Our general medial approach was to begin at the presacral space and obtain a connection with the plane of the lateral approach. Pelvic dissection was performed from the entrance of the pelvic cavity down to the pelvic floor. We could identify both the hypogastric nerve fibers and pelvic nerve by using high-definition 3D laparoscopy and preserve them. The IMV/left colic artery bundle was then carefully traced to the junction position from the IMA, and lymph node No.253 was dissected. The pelvic nerves and ureter were already carefully insulated, and the circumference of the IMA could be revealed. The mesocolon could be freed from the retroperitoneal position by anterior dissection. By gently applying a bipolar electrosurgical device, we dissected the SRA and blood vessels from the SRA to the intestinal wall and dissected lymph nodes No.252 and No.251. At this point, we had completed lymph node dissection and completely clarified the relationship between the LCA, IMV, IMA, SRA, and arteriae sigmoideae. Finally, we ligated the arteriae sigmoideae and vascular branch from the SRA into the intestinal wall (Fig. 3d) while preserving the left colonic vasculature (Fig. 3e). Energy devices and hemo-locks were used widely in this step.

After the above procedure was completed, we separated the rectal wall from the mesorectum with an adequate distance from the tumor in accordance with the T stage and position of the tumor using a harmonic vascular closure system. In order to provide enough space to insert an endoscopic linear stapler, we excised the mesorectum about 3–5 cm just underneath the rectal wall (Fig. 3f). Careful surgery was performed to avoid injury to the rectal wall and SRA, then the endoscopic linear stapler was fixed, the rectum was transected, and satisfactory TSME preservation of the left colic and superior rectal arteries was shown (Fig. 3g).

Lastly, a small 5-cm incision was made at the left lower abdomen, and the specimen was taken outside of the abdomen and transected. Intraabdominal presacral anastomosis was performed by double stapling techniques after inserting the anvil head of a 28-mm circular stapler into the oral side of the sigmoid colon. Double drains were placed, and no diverting stoma was performed.

In the TME group, the inferior mesenteric artery was severed at the root, the colon was severed 5 cm away, and digestive tract reconstruction methods were similar to the TSME group.

### Statistics

SPSS19.0 version was used for statistical analysis. Categorical variables were compared using a *χ*^2^ test. Continuous variables were presented as the mean (standard deviation) or median (range). These variables were compared using a Mann-Whitney *U* test. *P* values of < 0.05 were considered statistically significant.

## Results

The general characteristics of the included patients are listed in Table 1. There were 31 men (67.4%) and 15 women (32.6%) in the TSME group, and 81 men (58.7%) and 57 women (41.3%) in the TME group. The mean age was 64.05 ± 9.59 years and 63.50 ± 11.6 years in the TSME and TME groups, respectively. There were no significant differences in preoperative comorbidity, tumor size, depth of invasion, and lymph node metastasis between groups. The average distance between the tumor and anus of the TSME group was 11.20 ± 2.90 cm, and the distal margin was 5.44 ± 1.66 cm. The pathological stages of the patients for the TSME group were as follows: stage I, 21.7%; stage IIa, 2.2%; stage IIb, 47.8%; stage IIc, 6.5%; stage IIIa, 8.7%; and stage IIIb, 13%. The proportion of patients with normal preoperative carcinoembryonic antigen (CEA) was 58.7%. Approximately 19.6% of patients had CEA levels between 5 and 10 ng/ml, 17.4% of patients had CEA levels between 10 and 50 ng/ml, and only 2 patients had CEA levels > 100 ng/ml.

**Table 1:** Clinicopathological features between the TSME and TME groups

Factors	TSME, *n* = 46	TME, *n* = 138	*P* value
Age (years)	64.05 ± 9.59	63.50 ± 11.6	0.598
Gender			0.297
Male	31 (67.4%)	81 (58.7%)	
Female	15 (32.6%)	57 (41.3%)	
BMI (kg/m^2^)	22.59 ± 3.81	20.88 ± 4.33	0.588
Comorbidity			
Cardiovascular disease	10 (21.7%)	25 (18.2%)	0.603
Respiratory disease	3 (5.5%)	8 (5.8%)	0.858
Diabetes mellitus	9 (19.6%)	26 (18.2%)	0.930
Histological type			0.546
Differentiated type	32 (69.6%)	100 (72.5%)	
Undifferentiated type	14 (30.4%)	38 (27.5%)	
Tumor size (mm)	37.26 ± 14.75	36.62 ± 12.70	0.150
T category			0.482
T1	2 (4.3%)	19 (13.8%)	
T2	18 (39.1%)	50 (36.2%)	
T3	14 (30.4%)	39 (28.3%)	
T4	12 (26.1%)	30 (21.7%)	
N category			0.381
N0	9 (19.6%)	35 (25.4%)	
N1	30 (65.2%)	78 (56.5%)	
N2	7 (15.2%)	25 (18.1%)	
Conversion to open surgery	0	1 (0.7%)	0.559
Operation time (min)	218.56 ± 35.85	201.13 ± 42.65	0.004
Blood loss (ml)	25.76 ± 27.87	18.00 ± 24.91	0.997
Lymph node dissection	19.43 ± 9.46	18.03 ± 7.43	0.024

The operation time in the TSME group was longer than that in the TME group (218.56 ± 35.85 vs. 201.13 ± 42.65, *P* = 0.004; Table 1). Furthermore, the number of resected lymph nodes in the TSME group was greater than that in the TME group (19.43 ± 9.46 vs. 18.03 ± 7.43, *P* = 0.024; Table 1). The blood loss between groups was not significantly different (Table 1). The average hospital stay in the TSME group was a little shorter than that in the TME group (9.47 ± 2.02 days vs. 11.06 ± 7.61 days; Table 2).

**Table 2:** Postoperative complications

Factors	TSME, *N* = 46	TME, *N* = 138	*P* value
Postoperative hospital stay (days)	9.47 ± 2.02	11.06 ± 7.61	0.854
Mortality	0	0	1.000
Morbidity			0.128
Absent	42 (91.3%)	114 (82.6%)	
Present	4 (8.7%)	24 (17.4%)	
Anastomotic leakage	0	0	
Bleeding	0	1 (0.7%)	
Abdominal abscess	0	1 (0.7%)	
Ileus	0	1 (0.7%)	
Wound infection	2 (4.3%)	10 (7%)	
Anastomotic stenosis	0	0	
Urinary tract infection	1 (2.2%)	2 (1.4%)	
Ascites	1 (2.2%)	4 (2.8%)	
Urinary retention	0	2 (1.4%)	
Pneumonia	0	1 (0.7%)	
Cardiac-related complications	0	2 (1.4%)	

No mortality occurred in either group. One patient in the TME group underwent conversion to laparotomy due to bowel ischemia in the distal colon (Table 2). The total postoperative complication rates in the TSME and TME groups were 8.7% and 17.4%, respectively (Table 2). For severe complications between the two groups (anastomotic leakage and stenosis), the severity of complications was Clavien-Dindo classification grades 1–2, and there was no significant difference between groups.

## Discussion

In 1982, the British surgeon Heald proposed TME for rectal cancer and pointed out that the anatomical level of TME was clear, so that the operative quality can be assessed [7]. The main concerns were a higher anastomotic leakage rate, longer operative time, and higher blood loss after TME [8]. Lopez-Kostner et al. pointed out that TME was the standard operation performed for lower rectal cancers. TME is not necessary for cancers of the upper rectum [2]. Therefore, the TSME technique was introduced to achieve satisfactory local control and low morbidity. Partial mesorectal excision is applied in TSME [9].

According to Willian’s report in 1983, only 6% of patients had distal intraluminal diffusion > 2 cm [10]. Pollett and Nicholls observed that there were no differences in the local recurrence rate of rectal cancer between distal margins < 2 cm, 2–5 cm, and > 5 cm [11]. A randomized prospective study of NSABB (the National Surgical Adjust-Burst and Bowel Project) showed that the local recurrence rate was not significantly different between distal rectal margins < 2 cm, 2–2.9 cm, and > 3 cm [12]. According to the Practice Parameters for the Management of Rectal Cancer (2013 edition), a 2-cm distal margin is more acceptable than 5 cm, but a 5-cm distal margin is still recommended. Total mesorectal resection (TME) should be used for tumors located in the middle and lower two-thirds of the rectum, regardless of whether it is performed with low anterior resection (LAR) or combined abdominal and perineal resection (APR). For tumors in the upper one-third of the rectum, resection of the mesentery can be carried out according to the tumor situation, and the distance between the distal margin and tumor should be > 5 cm. The recommended grade was 1A [13].

TME was performed according to the distance between the distal margin of the rectal tumor and anus < 10 cm, while TSME was performed for patients with a distance between the distal end of the rectal tumor and anus of 10–15 cm in the author’s medical department.

Oncological outcomes after surgery can be divided into two aspects: long-term survival and local recurrence rate. Law et al. [14] reviewed 622 patients. The 5-year local recurrence rate for TME and partial mesorectal excision (PME) for proximal cancer was 10.7% and 7.4%, respectively. The disease stage was associated with a higher risk of local recurrence. There was no difference in the local recurrence rates of TME and PME. The 5-year cancer-specific survival rates with and without TME were similar at 74.0% and 76.1%, respectively. Kim et al. [15] reported that the 5-year cancer-specific survival rate was 77.5% and the local recurrence rate was 9.2%, with 782 cases of rectal cancer after TSME with pathologic stages I–III. The risk factors affecting cancer-specific survival rate were the pT stage, pN stage, positive distal resection margin, and positive circumferential resection margin. The risk factors affecting local recurrence were the pN stage, positive distal resection margin, and positive circumferential resection margin. Another study from a Korean reviewed experience in 1276 patients with rectal cancer showed that the overall local recurrence rate was 5.4%. The 5-year local recurrence rates were 3.8%, 4.7%, and 8.4% in stages I, II, and III, respectively. The 5-year cancer-specific survival rates were 93.8%, 84.5%, and 64.5% in stages I, II, and III, respectively. The risk factors were the pN stage and circumferential resection margin [16]. Zakir et al. [17] performed an analysis with 11 years of experience in 1063 rectal cancer patients who underwent laparoscopic and open TSME surgery. The 5-year local recurrence rate was 7.1%. The overall 5-year and cancer-specific survival rates were 66.8% and 76.0%, respectively. There was no difference in the local recurrence rate between laparoscopic or open resection. The overall and cancer-specific survival rates were 72.8% and 80.1% in the laparoscopic surgery group, and 62.9% and 73.1% in the open surgery group, respectively. The results showed that laparoscopic surgery was better than open surgery in overall and cancer-specific survival. There was no difference in survival in patients with stage I. However, the survival rates in patients with stages II and III among the laparoscopic surgery group were better than those in the open surgery group, which shows the superiority of laparoscopic TSME surgery for the long-term prognosis of rectal cancer.

Korean scholars conducted a study on the safety and prognosis of TSME after neoadjuvant chemotherapy for rectal cancer. Patients received 5-FU with leucovorin chemotherapy and radiotherapy (5040 cGy) for 25 cycles. TSME was performed 4–6 weeks later. The results showed that the overall complication rate was 9.6%, empirical leadership was 2.6%, internal construction was 2.6%, the 5-year survival rate was 58.1%, and the 5-year disease-free survival was 2.6% [18]. At present, China, South Korea, and the USA have formulated similar guidelines for preoperative radiotherapy and chemotherapy for middle and low rectal cancer, but there is no specific reference data for preoperative radiotherapy and chemotherapy for upper rectal cancer. The purpose of this paper is to introduce a new method of TSME and discuss the safety of the operation. Long-term survival and local recurrence have not been discussed.

TSME surgery based on TME is now accepted as a standard for rectal cancer surgery, and laparoscopic rectal cancer resection is accepted widely in the world even though it is a challenging procedure for surgery. Blood loss in the laparoscopic group is well shown, with an average of 90 to 320 ml [19]. The average blood loss in our study was 25 ml lower than that reported in the literature. We can identify neurovascular lesions using high-definition 3D laparoscopy to preserve them, and we use a bipolar electrosurgical device to reduce injury, which is beneficial for accurate operation.

The overall complication rate in laparoscopic TSME operation was lower than that in the open operation group. The rate of anastomotic leak showed no statistical difference between the two operation methods. The average leak rate for rectal cancers was 10% [20].

Zakir et al. [17] reported that the overall complication rate was 29.8% in TSME for rectal cancer patients. The rate of anastomotic leakage was 3.87% in the open TSME group and 2.97% in the laparoscopic TSME group. There was no statistical difference between groups. In our study, the incidence rate of postoperative anastomotic leakage was 0%. Three patients had complications after surgery, and the overall complication rate was 6.5%. The three complications were wound infection, fluid collection, and urinary retention with a Clavien-Dindo grading of 1–2. Yoo et al. [20] evaluated the optimal duration of urinary catheterization after TSME for rectal cancer. Logistic regression analysis was performed to determine the risk factors for urinary retention. The variables including age, sex, ASA grade, surgical procedure, TNM stage, tumor position, preoperative radiotherapy, duration of urinary catheterization, and time of surgery were not significant risk factors for urinary retention.

At present, a 3D laparoscopic system (Aesculap German) is used in laparoscopic surgery in our department. Single and reduced port laparoscopic surgery, robot operations, and Ta-TME operations are not used for TSME. The surgeons who performed TSME had more than 10 years of experience in gastroenterostomy and had experience with open TSME. The difficulty of the TSME operation is the management of the mesorectum. Seiji [21] has reported on the management of the mesorectum in the narrow pelvis, which our treatment method is based on. First, the right part of the mesorectum is lifted from the right side of the sigmoid mesocolon to expose the inferior mesenteric artery and vein, left colonic vessels, sigmoid colonic vessels, and superior rectum vessels. The assistant lifts the left mesentery of the sigmoid colon, exposes the above vessels, expands the sigmoid mesocolon again, penetrates the mesentery from the right side, and exposes the surrounding vessels. Expansion of the pelvic cavity along the vessels is continued, and the mesorectum is repaired from the left to the right side 4–5 cm above the tumor. According to the location of the tumor, the branches of the severed vessels are determined and 2–3 cm of the intestinal wall is repaired. The rectum is dissected using an endo-GIA stapler.

Laparoscopic TSME has been used for rectal cancer and can obtain satisfactory functional results compared to open resection and TME. We do not think that the reduction in the hospital stay is due to the acceleration of the intervention, as per Enhanced Recovery After Surgery (ERAS), but is due to an increase in the doctors’ confidence in reducing the risk of postoperative complications after vascular preservation. Three-dimensional CT-A examination is important for the preoperative evaluation of sigmoid colon vascular classification and intraoperative management of the sigmoid and left colon vessels. However, preoperative examination could not obtain information on the traffic branch. The biggest advantage of this operation is the maintenance of the blood supply of the proximal and distal intestines and the sufficient length of the intestine, so there is no need for temporary defunctioning stoma. Temporary defunctioning stoma only increases the complexity of the operation, and closure of the temporary stoma increases the risk of complications. In addition, the results of the statistical analysis showed that the number of lymph nodes in the TSME group was greater than that in the TME group. It cannot be concluded that TSME was significantly better than TME for lymph node dissection, suggesting that TSME was not inferior to TME.

## Conclusions

Laparoscopic TSME with preserved left colic and superior rectal arteries is a technically challenging procedure. Intact visceral pelvic fibro is protected with even greater accuracy than other techniques by 3D laparoscopy, which offers an optimal vision. TSME with preserved left colic and superior rectum arteries did not increase the risk of operation compared with TME but increased the surgeon’ s confidence in patient outcomes. Therefore, laparoscopic TSME with preserved left colic and superior rectal arteries can be safely performed for rectal cancer patients as an alternative to TME.

## Data Availability

All experimental data used to support these findings are included in the article.

## Abbreviations

TME: Total mesorectal excision
TSME: Tumor-specific mesorectal excision
IMA: Inferior mesenteric artery
IMV: Inferior mesenteric vein
SRA: Superior rectal artery
NVB: Neurovascular bundle
PME: Partial mesorectal excision
